# Liquid Crystal-Templated Porous Microparticles via Photopolymerization of Temperature-Induced Droplets in a Binary Liquid Mixture

**DOI:** 10.1021/acsomega.3c00490

**Published:** 2023-05-30

**Authors:** Mehzabin Patel, Alberto Alvarez-Fernandez, Maximiliano Jara Fornerod, Anand N. P. Radhakrishnan, Alaric Taylor, Singg Ten Chua, Silvia Vignolini, Benjamin Schmidt-Hansberg, Alexander Iles, Stefan Guldin

**Affiliations:** † Department of Chemical Engineering, University College London, London, WC1E 7JE, United Kingdom; ‡ Yusuf Hamied Department of Chemistry, University of Cambridge, Cambridge, CB2 1EW, United Kingdom; ¶ Chemical & Process Engineering, Coating & Film Processing, BASF SE, 67056 Ludwigshafen am Rhein, Germany; § Lab-on-a-Chip Research Group, Department of Chemistry and Biochemistry, University of Hull, Hull, HU6 7RX, United Kingdom

## Abstract

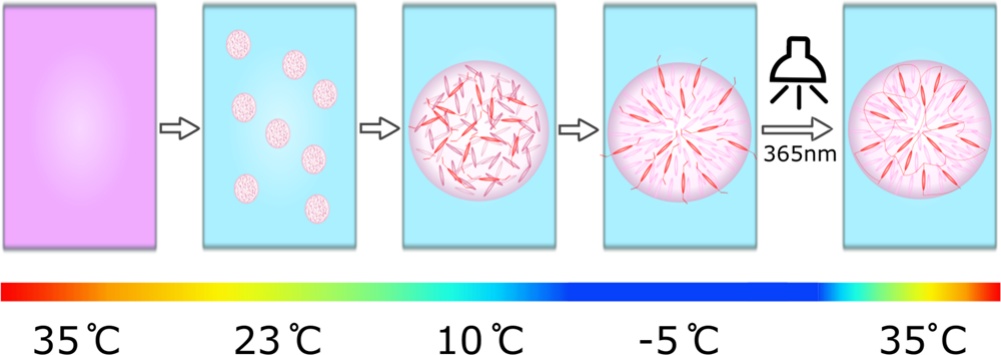

Porous polymeric microspheres are an emerging class of materials, offering stimuli-responsive cargo uptake and release. Herein, we describe a new approach to fabricate porous microspheres based on temperature-induced droplet formation and light-induced polymerization. Microparticles were prepared by exploiting the partial miscibility of a thermotropic liquid crystal (LC) mixture composed of 4-cyano-4′-pentylbiphenyl (5CB, unreactive mesogens) with 2-methyl-1,4-phenylene bis4-[3-(acryloyloxy)propoxy] benzoate (RM257, reactive mesogens) in methanol (MeOH). Isotropic 5CB/RM257-rich droplets were generated by cooling below the binodal curve (20 °C), and the isotropic-to-nematic transition occurred after cooling below 0 °C. The resulting 5CB/RM257-rich droplets with radial configuration were subsequently polymerized under UV light, resulting in nematic microparticles. Upon heating the mixture, the 5CB mesogens underwent a nematic–isotropic transition and eventually became homogeneous with MeOH, while the polymerized RM257 preserved its radial configuration. Repeated cycles of cooling and heating resulted in swelling and shrinking of the porous microparticles. The use of a reversible materials templating approach to obtain porous microparticles provides new insights into binary liquid manipulation and potential for microparticle production.

## 1 Introduction

Micrometer-sized polymeric particles have attracted interest in applications such as optical displays,^1,2^ drug delivery,^3,4^ chemical separation,^5−7^ emulsion stabilization,^8^ and catalysis.^9^ Material platforms based on liquid crystals offer complex internal architectures and unique optical properties.^10−12^ As an example, difunctional mesogenic monomers have been used to form liquid crystal elastomer droplets/shells, which possess the ability to undergo changes in shape in response to external stimuli.^13^ Several studies have demonstrated their promising applications as actuators, for example, their unique suitability as artificial muscles,^14−17^ and their potential as micropumps.^18−20^ Furthermore, the long-range order and fluidic properties of liquid crystals confined in a droplet can serve as a template for polymerization reactions which result in anisotropic polymeric particles with distinct shapes and configurations.^21−24^ Such liquid crystal droplets often contain a mixture of reactive (polymerizable) and nonreactive (nonpolymerizable) thermotropic mesogens with tunable configurations. Typically, the reactive mesogens are photopolymerized, and the nonreactive mesogens are extracted, leaving micrometer-sized anisotropic polymeric particles with distinct shapes and complex internal structures.^25^ Nanoporosity of the microparticles can be tuned,^24^ and “patchy particles” have been developed where colloids are positioned at the defects of the liquid crystal droplet.^22,26^

There are several approaches for the formation, control, and stabilization of liquid crystal microdroplets including vortexing,^27,28^ high-pressure homogenization,^22^ sonication,^29,30^ emulsifying liquid crystal in glycerol,^22,24,25^ encapsulation in a polymeric capsule,^31^ microfluidics,^32−35^ and dispersion polymerization.^36,37^ We have recently established a new route to liquid crystal microdroplets based on temperature-induced formation in a partially miscible liquid mixture composed of cyano-4′-pentyl-biphenyl (5CB) and methanol (MeOH).^38,39^ Regular binary liquid mixtures exhibit a miscibility gap for certain compositions and temperature ranges. The demixing of these liquids takes place by two pathways: *nucleation* - when a concentration partitioning reaches a critical size, droplets grow in a new phase and propagate; and *spinodal decomposition* - where the mixed phase spontaneously separates into distinct domains.^40−42^ In the study by Patel et al., droplet size and number of nucleations were controlled by cooling rate and quench depth.^39^ Droplet formation by nucleation in a binary liquid mixture undergoes a series of stages of growth from nucleation to growth by diffusion, coalescence, and Ostwald ripening.^43^ Arresting this phase separation process at one of these stages opens up a new avenue for the temperature-controlled formation of stable droplets. There are also some notable examples of binary fluid systems forming networks and structures. Clegg et al. utilized this method with colloidal particles in a partially miscible mixture to form cellular networks.^44^ Colloidal particles have also been used to arrest the demixing of a partially miscible liquid by spinodal decomposition to form bicontinuous interfacially jammed emulsion gels (bijels).^45^ Another concept to stabilized particles is the formation of layered multishells by liquid–liquid phase separation of ternary mixtures in droplets assembled by microfluidics,^46^ which has recently been exploited for colloids with photonic stop band^35^ as well as to fabricate polymer-based microcapsules.^47^

The formation of microparticles by temperature-induced droplet formation and light-induced polymerization is a novel concept. First, it utilizes the natural demixing of a binary liquid by temperature control to form droplets. Second, it employs photopolymerization of the reactive mesogens and thereafter extraction of nonreactive mesogens, thereby exploiting a reversible templating process to create a microparticle product, which has the ability to undergo shape change by swelling and shrinking in response to external stimuli. In this work, we incorporate the reactive mesogen 2-methyl-1,4-phenylene bis-4-[3-(acryloyloxy)propoxy]benzoate (RM257) in the binary system mentioned above for the production of liquid crystal microparticles. We study the cooling-induced formation of 5CB/RM257-enriched droplets in methanol, photopolymerize them in their nematic phase and further investigate the polymeric microparticles. We use optical cross-polarized microscopy to quantify phase and shape of the particles, and provide a descriptive analysis of the microparticle texture via scanning helium ion microscopy (SHIM).

## 2 Experimental Section

### Reagents

The liquid crystal 4-cyano-4′-pentylbiphenyl (5CB) was obtained from Synthon Chemicals (99.5% (GC)). 2-Methyl-1,4-phenylene bis-4-[3-(acryloyloxy)propoxy]benzoate (RM257) was acquired from Apollo Scientific. Dichloromethane (99%, puriss) (DCM), MeOH (HPLC grade), acetone (99.8% Chromasolv), and 1-hydroxycyclohexyl phenyl ketone were purchased from Sigma-Aldrich. All compounds were used without further purification.

### Sample Preparation

5CB and RM257 (20%) were placed in a glass vial and mixed by vortex and heated until the mixture was clear. DCM was added to the vial on a hot plate and stirred at 60 °C overnight. The mixture was then placed in a vacuum oven at 30 °C for 1 h. Photoinitiator 1-hydroxycyclohexyl phenyl ketone (1%) was added and mixed at room temperature for 3 h. Samples containing the 5CB/RM257 blend and MeOH were mixed in a glass cuvette, which was enclosed by a Peltier-regulated sample compartment that allowed control over both temperature and stirring (Quantum Northwest, Qpod 2e). Unless stated otherwise, a 30:70 volume ratio of 5CB/RM257:MeOH was used for all experiments. Samples were heated to 40 °C in the cuvette.

### Sample Analysis

For microscopic analysis, 10 μL of the homogeneous 5CB/RM257:MeOH mixture was deposited between a glass slide and coverslip and sealed with varnish (purchased from Rimmel: 15–40% ethyl acetate, 15–40% butyl acetate, 5–15% nitrocellulose, 1–10% isopropylalcohol) to prevent MeOH evaporation. The slide was placed under an upright microscope (Zeiss, Axio Scope A1), that was operated in transmission mode. Crossed polarizers were used to observe anisotropic behavior. A λ (550 nm) waveplate served to introduce a fixed amount of retardation between the ordinary (*n*_*o*_) and extraordinary (*n*_*e*_) rays passing through the LC and quantify the birefringence using the Michel-Levy chart. A temperature controlled sample stage (Linkam, LTS120) was used for all experiments. Nitrogen was introduced into the chamber to prevent condensation at low temperatures. In situ droplet formation and growth were recorded by time-resolved digital image acquisition using a Lumenera Infinity 3–3UR camera with a resolution of 1936 × 1456 pixels.

Photopolymerization of the liquid crystals was performed with a Prior Lumen 200 UV lamp that delivered 90 mW cm^–2^ through illumination from the top-side of the optical microscope objective using a 365 nm excitation filter, while allowing simultaneous viewing of the process with transmitted light. The liquid crystal droplets were exposed to UV light for 5 s to 5 min.

For extraction and analysis of droplets, the above experiment was carried out in a microfluidic glass chip with a 35 × 5 × 0.03 mm chamber.^48^ The microchip was fabricated from two layers of Schott B270 glass 3 mm base and 1 mm top layer. These precoated glass wafers were exposed to UV with a mask to create the pattern for etching. The glass was etched with buffered HF solution to create the chamber. Then, a Datron 7 CNC milling machine was used to create the access holes, and this was thermally bonded to the etched bottom layer. The top layer featured 1.5 mm diameter inlet and outlet holes. PTFE tubing was glued on each end and used to insert the homogeneous 5CB/RM257 and methanol mixture into the channel. Droplet formation and exposure to UV light was as described above. Extraction of the polymer was carried out by flushing the channel with acetone. The particles were washed twice by centrifugation (14500 rpm, 15 min) and redispersed into acetone. 5CB removal was confirmed using an FTIR spectrometer VERTEX 70/70v (Bruker Corporation, Germany) coupled with Platinum Diamond ATR. Image analysis of the particles was done by optical microscopy and a Helium Ion Microscope (Orion Nanofab, Carl Zeiss) (SHIM) at an accelaration voltage of 25 kV.

Quantitative droplet investigation was carried out by computational image analysis code developed in Python^39,49^ and using Fiji,^50^ ImageJ.^51^

Birefringence measurements were calculated using the Michel-Levy chart. Droplets were assumed to be spherical and thus of equal thickness. The observed highest order interference colors displayed by the droplets under cross-polarized light was compared to the Michel-Levy chart. The diagonal line crossing the ordinate section of the color and thickness were used to determine birefringence.

## 3 Results and Discussion

**Figure 1:**
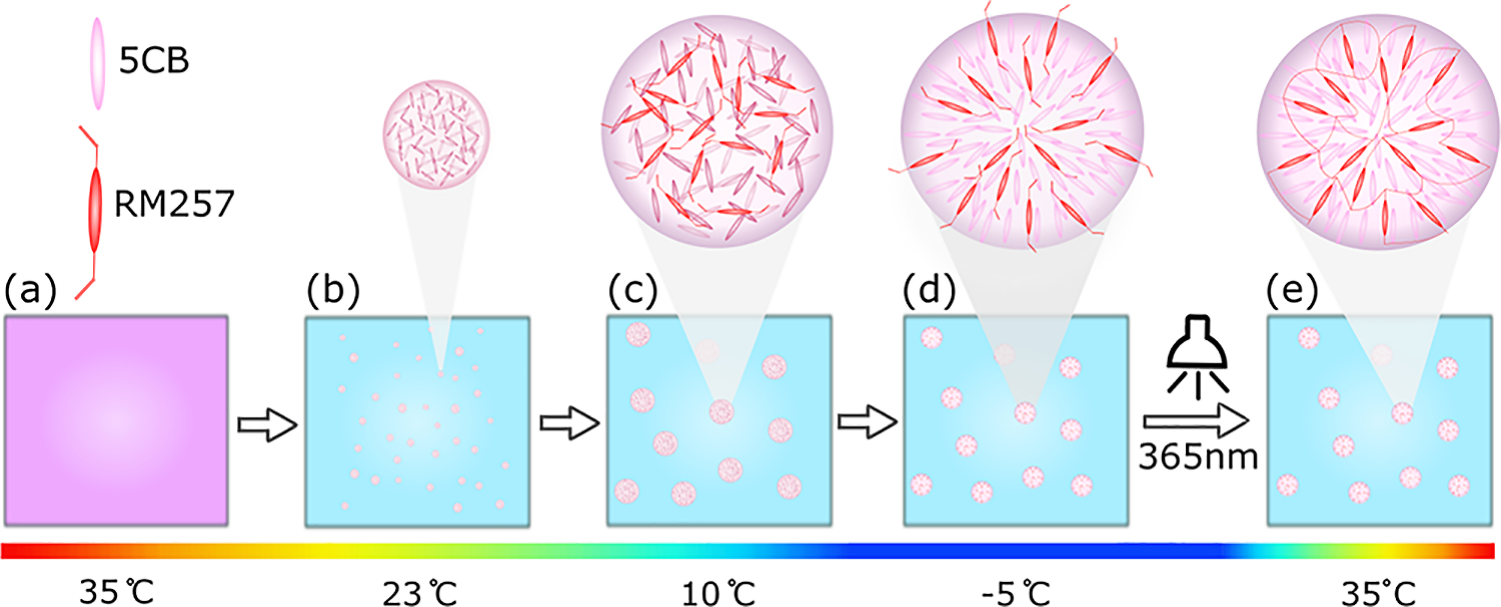
Schematic illustrations of the porous microparticle formation process. (a) A homogeneous mixture of 70 vol % methanol +30 vol % 5CB/RM257 is prepared at 35 °C, i.e., above the upper critical solution temperature. (b) Isotropic liquid crystal enriched droplets are nucleated at 23 °C, which (c) continue to grow during further cooling. (d) Cooling below 0 °C triggers isotropic-to-nematic phase transition of the 5CB/RM257-rich droplet. (e) Subsequent exposure to UV light polymerizes the photoreactive RM257 mesogens, providing a porous structural matrix that is suitable for extraction while no longer being temperature-sensitive.

The liquid crystal used in these experiments was a mixture of 5CB, which contains nonreactive mesogens and RM257, serving as the reactive monomer at 20 wt/wt % (see Supporting Information, Figure S1). Multicomponent liquid crystals are commonly used to tailor their properties,^52^ such as clearing point. In the following experiments, 5CB and RM257 mixtures are treated as one single component of a binary mixture. Enriched droplets of the liquid crystal mixture 5CB/RM257 were formed by cooling a mixture of 30 vol % 5CB/RM257 with 70 vol % methanol in a sealed compartment from 35 °C to −5 °C at 20 °C min^–1^ as introduced by Patel et al.^39^ and illustrated in Figure 1a-d. The focus in this previous study was on the reversible formation of 5CB-enriched isotropic microdroplets that were size-tunable in the range from 4 to 12 μm via controlling the quench depth and could be subsequently converted to nematic droplets via further cooling below the isotropic-to-nematic transition temperature. Herein, we exploit this phenomena for the formation of microdroplets but then convert them into microparticles via photopolymerization.

**Figure 2:**
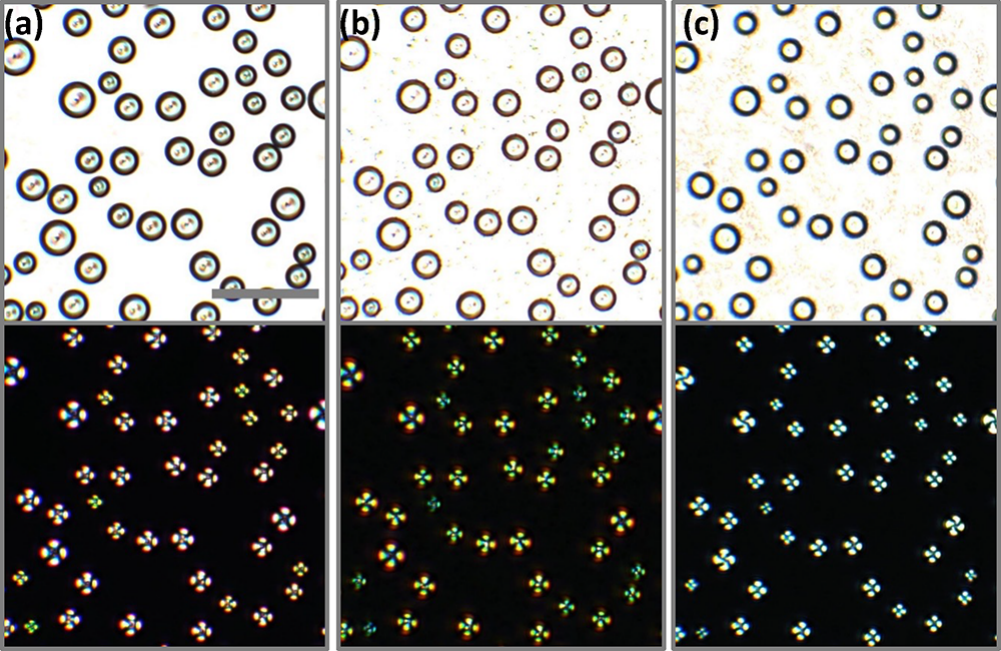
Liquid crystal templated microparticles at different stages of the formation process under transmitted light. The same field of view images were taken under brightfield illumination (top) and crossed-polarized light (bottom). (a) 5CB/RM257-rich droplets cooled to −5 °C. (b) 5CB/RM257-rich droplets at −5 °C after photopolymerization. (c) RM257 polymer particles after heating to 35 °C. Scale: 50 μm.

Droplets were imaged under an optical microscope in transmission mode. Imaging began at 35 °C. For the herein studied mixture of 30 vol % 5CB/RM257 with 70 vol % methanol, phase separation was observed at a temperature of 23 °C without hysteresis. This phase separation temperature will be referred to as *T*_*ps*_ in the following discussion. 5CB/RM257-enriched isotropic droplets formed by nucleation and subsequently grew. The droplets underwent an isotropic-to-nematic transition at 0 °C and formed nematic micrometer-sized droplets with a radial configuration, as shown in Figure 2a. Deploying the herein described experimental procedure, we consistently observed radial configuration. However, in a related study following the long-term phase separation dynamics of 5CB-enriched microdroplets obtained from binary fluid mixtures, we witnessed defect migration of non-cross-linked droplets over time toward escaped-radial configuration, offering a route to alternative configurations.^53^

We note that as a consequence of the presence of RM257, the observed phase separation temperature and isotropic–nematic transition occurred at approximately 1 °C higher than that described by the binary phase diagram for binary liquid mixtures of pure 5CB and MeOH.^38^ A full phase diagram for this new system has not been mapped, and this change in phase separation/transition is only for the observed volume ratio in comparison to the binary system. In the absence of RM257, 5CB-enriched droplets were also found to form radial droplets in methanol.^39^ In comparison, previous studies show that 5CB droplets dispersed in water generated bipolar configurations and for 20 wt/wt % RM257/5CB mixtures axial configurations were formed.^24^ These differences in liquid crystal configuration have been ascribed to its dependence on the elastic constants and/or surface anchoring of the nematic mixture,^24,54,55^ which will differ in a partially miscible liquid mixture.

With the addition of the photoinitiator, when 5CB/RM257-rich droplets were exposed to UV light (365 nm), the RM257 mesogens formed a highly mixed polymer network swollen with nonreactive 5CB mesogens. Previous research has demonstrated that the anisotropic internal ordering of the droplet is governed by the 5CB, and polymerization occurs with minimal perturbation to the original alignment of the nonpolymerized matrix.^14,22,24,56,57^ The optimal length of exposure time to UV light was determined by measuring the shrinkage of the particles upon heating above *T*_ps_. Exposure for 300 s resulted in no measurable changes to particle diameter before and after heating (see Supporting Information Figure S2). When droplets were exposed for less than 300 s, there was a reduction in particle diameter upon heating, suggesting that the RM257 had not fully polymerized. We note that photopolymerization of the liquid crystal droplets-rich preserved the nematic droplet radial configuration even when shrinkage occurred. With a 300 s exposure time, both the configuration and size were preserved. Figure 2 shows 5CB/RM257-rich droplets before (a) and after (b) photopolymerization, and upon heating from −5 to 35 °C (c). In the absence of reactive monomer RM257, 5CB-rich droplets in methanol would follow a fully reversible process, whereby 5CB droplets would undergo a nematic–isotropic transition upon heating to −1 °C, and the droplets would disappear back into the methanol upon further heating above *T*_ps_. With the addition of the reactive monomer RM257 and after exposure to UV light, polymerized particles remained intact upon heating above *T*_ps_, as shown in Figure 2c.

**Figure 3:**
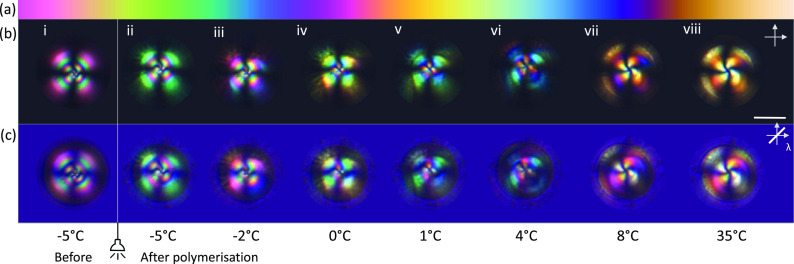
Birefringence of liquid crystal-templated microparticles. (a) Colors of the Michel-Levy chart decreasing from third order (left) to first order (right) interference colors. (b) Evolution of a liquid crystal particle with increasing temperature after photopolymerization between crossed polarizers and (c) between crossed polarizers with a λ (550 nm) plate inserted. Scale: 10 μm.

The birefringence (Δ*n* = *n*_*e*_ – *n*_*o*_) of 5CB/RM257-rich droplets before and after photopolymerization was investigated using polarized optical microscopy. Figure 3a displays the interference colors of the Michel-Levy chart, with the order of interference colors decreasing from left to right.^58^Figure 3b and c show the evolution of a single 5CB/RM257-rich droplet between crossed-polarizers and with a first order retardation λ plate inserted into the optical train, respectively. After cooling the 5CB/RM257:MeOH mixture to −5 °C, the nematic droplet displayed third order interference on the edges of the droplet with the order of interference colors seen in the isochromes decreasing toward the melatope, as shown in Figure 3b(i). Upon photopolymerization at −5 °C, the birefringence had reduced slightly (Figure 3b(ii)) and began to display second order interference colors as the droplets were heated (Figure 3b(iii-vi)). At 35 °C, first order interference colors could be observed (Figure 3b(viii)). When a λ plate was inserted, a relative retardation of exactly one wavelength (550 nm) was introduced between the ordinary (*n*_o_) and extraordinary (*n*_e_) wavefronts, and a magenta color was observed, as shown in Figure 3c. At 35 °C, the optical path difference increased for the northeast and southwest quadrants of the particle, as the color shifted to higher order interference, while in the northwest and southeast quadrants, the optical path difference decreased as the color shifted to lower first order interference. Therefore, the particles showed a radial configuration with positive uniaxial birefringence (Δ*n*).

**Figure 4:**
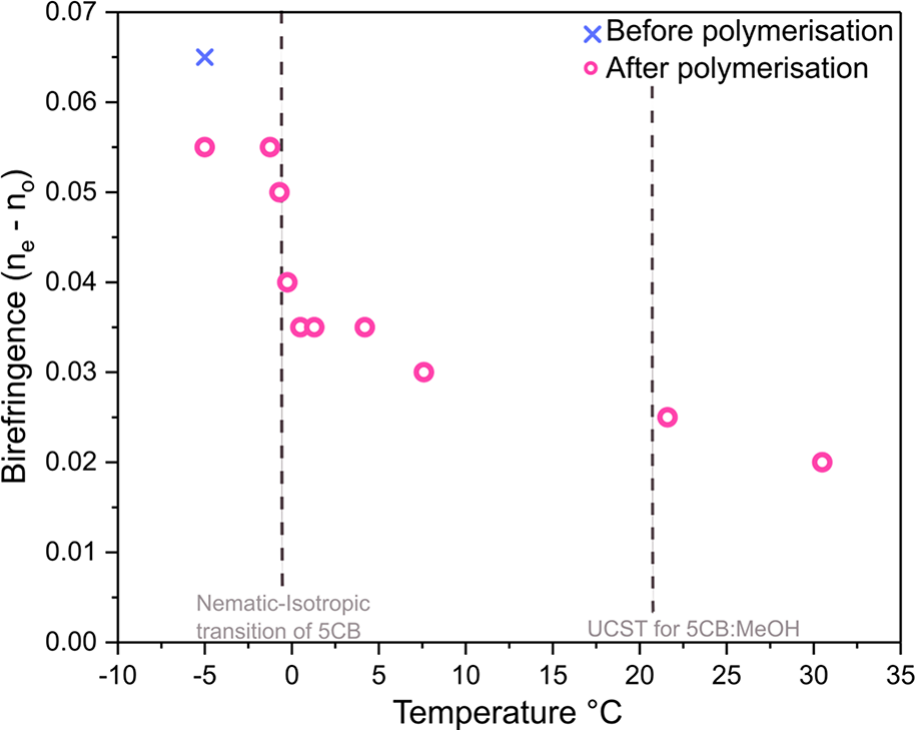
Birefringence measurements of particles. Birefringence values of as a function of temperature from −5 to 35 °C.

Quantitative evaluation of birefringence as a function of temperature was performed using the Michel-Levy chart and the diameter of the particles, which were assumed to be spherical and thus of equal thickness. The results displayed in Figure 4 indicate a decrease in birefringence (Δ*n*) with increasing temperature. The decrease in birefringence relates to the decreasing order parameter of the liquid crystal. With the RM257 arrested in a photopolymerized network, changes in the birefringence are likely related to the 5CB mesogens, either as a consequence of a reduction in their nematic director field or due to mesogen diffusion out of the microparticle. Over 50% of the birefringence reduction occurred between −5 and 5 °C. A small increase in nematic–isotropic transition temperature of 5CB was to be expected here as the polymer network confined the nematic phase of the 5CB, thus increasing its stability.^59^ Between 5 and 35 °C, the birefringence continued to decrease, albeit at a slower rate. This may be either due to trapped 5CB molecules within the polymeric network, which were able to retain their nematic order well above the isotropic–nematic transition temperature and only eventually become isotropic or due to 5CB molecules escaping the polymer particle and becoming homogeneous with methanol at high temperatures, suggesting that the particle was porous.

**Figure 5:**
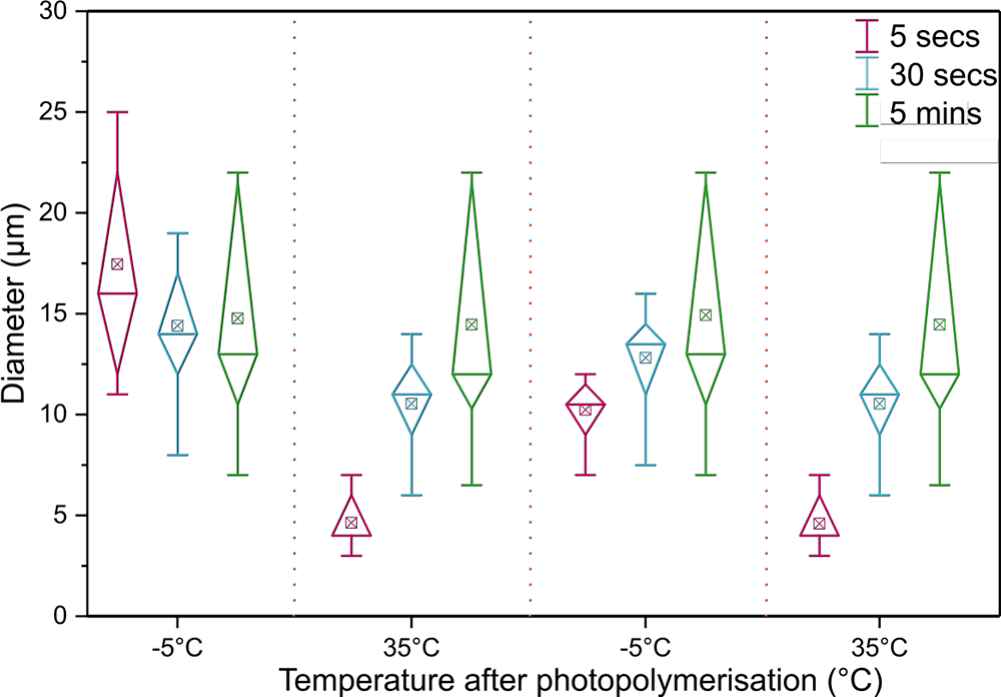
Swelling and shrinking of microparticles. Diameter of microparticles as a function of temperature over two cooling and heating sequences. Color notation indicated different lengths of UV exposure for photopolymerization (5 s, 30 s, and 5 min).

The hypothesis that the particles were porous was further supported in subsequent cooling and heating cycles, and shown in Supporting Information, Figure S3. Upon cooling to −5 °C, new 5CB-rich droplets nucleated outside of the particles and isotropic–nematic transition of these droplets occurred at −1 °C, indicating that a substantial amount of 5CB mesogens had left the particles during the previous heating cycle. Furthermore, 5CB-rich droplets also nucleated inside the microparticle, which became swollen upon cooling to −5 °C in comparison to 35 °C. Between crossed-polarizers, nematic order of the new droplets could be seen. However, the clear radial configuration of the microparticles could no longer be observed due to the birefringence of the new nematic 5CB droplets appearing in the pores of the particles. See Supporting Information, Figure S3 (1c and 2c). These factors are all consistent with a porous nature of the polymeric particles, which were also found to shrink with heating and swell with cooling, likely as a consequence of the outgoing and incoming flux of 5CB mesogens. This behavior was observed for all UV exposure times, as shown in Figure 5.

Figure 5 is a box and whisker plot, which presents the variation in diameter of droplets by displaying the data distribution through their quartiles. The line splitting the box represents the median, and the points of the box on either side the lower and upper quartile values, respectively. In particular, particles with low exposure times exhibited the most pronounced shrinkage of droplets upon heating and would swell by up to 50% when cooled to −5 °C. Particles with a higher exposure times shrunk or swelled to a much lesser degree. This swelling and shrinking action is shown for two consecutive rounds in Figure 5, but could be repeated over many more cycles without showing deterioration. A comparison of the effect of UV exposure time on shrinking and swelling of particles is shown in Supporting Information, Figure S3.

Finally, the particles were extracted by repeating the experiments described above in a microfluidic glass chip with a single chamber of diameter = 5 mm, length = 35 mm, and depth = 0.03 mm (see Supporting Information, Figure S4). A homogeneous mixture of 5CB/RM27:MeOH above *T*_ps_ was administered into the microfluidic chip that was placed on a temperature-controlled stage. Cooling and photopolymerization were performed in the same manner as described previously, and the particles were extracted by flushing the chamber with acetone.

**Figure 6:**
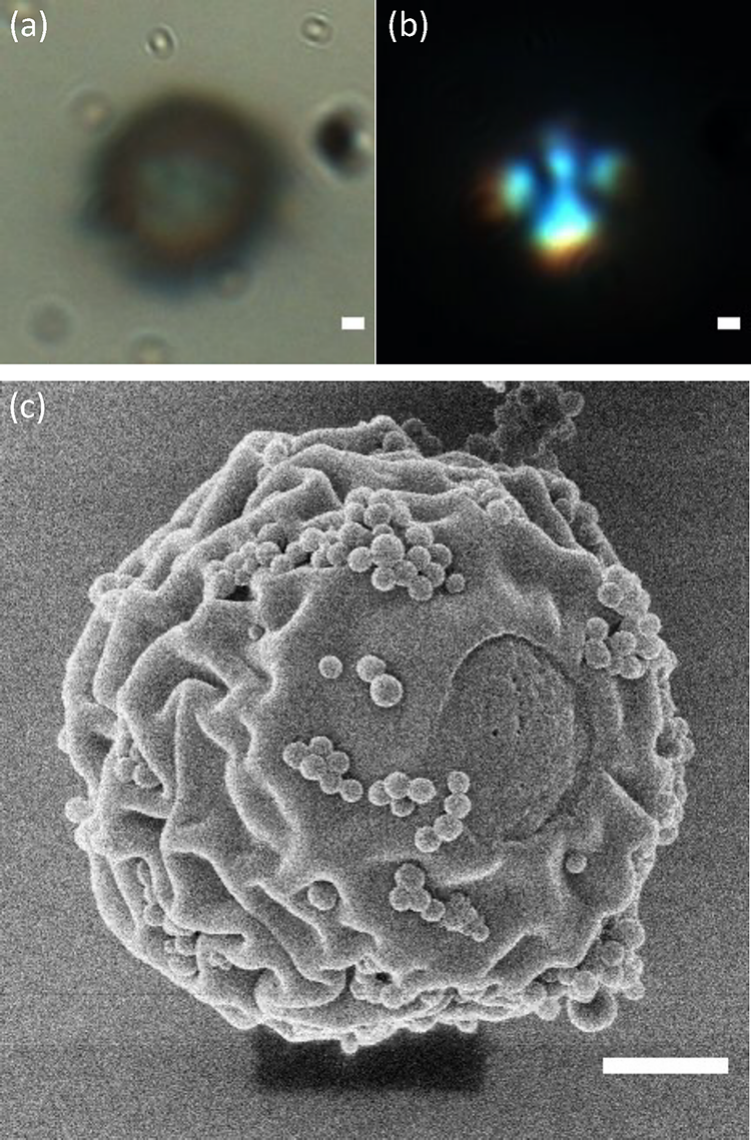
Microparticle extraction. Polymeric microparticle templated from radial droplets in a binary liquid imaged under (a) brightfield light, (b) crossed polarizers, and (c) SHIM. Scale: 1 μm.

The 5CB was removed from the particles by washing with acetone and centrifugation. 5CB removal was confirmed by IR analysis after each wash (see Supporting Information, Figure S5), and the particles were analyzed by optical microscopy and scanning helium ion microscopy (SHIM). Figure 6 presents the same polymer microparticle with different imaging techniques. Under optical microscope with crossed polarizers, they were still birefringent (Figure 6b) and had a diameter range of 1–10 μm. SHIM images presented in Figure 6c show the particle had a wrinkled texture with 200 nm particles attached to its surface. Other SHIM particles can be seen in Supporting Information, Figure S6. The wrinkled surface indicates some degree of shrinkage of the particle after extraction of 5CB, and the presence of smaller particles on the surface is in line with secondary nucleation and photopolymerization of RM257 from the continuous phase. Particles close to or touching each other at the time of UV exposure, fused together at their point of contact during photopolymerization, while still maintaining their individual radial configurations (see Supporting Information, Figure S6).

A notable observation was the presence of a crater or a tear on the surface of each particle. This may be related to polymerization-induced phase separation triggered upon UV irradiation, giving rise to phase separation in the ternary liquid and thus the formation of a concentric shell structure of RM257-enriched and RM257-depleted regions.^35,46,47^ The buildup of mechanical stress related to polymerization-induced shrinkage of the outer shell and the enclosure of incompressible nonreacting liquid (5CB) may cause the polymer shell to bulge out and eventually break (either during polymerization or solvent rinse), which was observed in a related study.^60^

## 4 Conclusion

We report the synthesis of polymeric porous microparticles from phase separation of binary liquid mixtures. Nematic 5CB/RM257-rich droplets with radial configurations were formed by cooling and polymerized by exposure to UV light (365 nm). Heating after polymerization led to a reduction of the mesogen director field component and eventual escape of 5CB mesogens from the polymerized microparticle as it reached *T*_ps_ and became miscible with methanol. Particles were found to shrink and swell with heating and cooling due to their porous nature, as 5CB was able to enter and leave particles according to temperature change. The microparticles maintained the optical properties of nematic 5CB, retaining its radial configuration and had a different morphology on the outer surface compared to the inner surface of the particle, creating an asymmetric particle shape. The generation of porous microparticles from bulk via temperature manipulation of binary liquid mixtures provides a promising platform for porous microparticle production. Furthermore, their anisotropic nature and ability to adapt their shape to temperature stimuli offers new opportunities for applications as actuators and microelectromechanical systems.

## Supporting Information Available

Western blot methodology; Characterization of SEC fraction isolates; Equivalent circuit model evaluation; Goodness of fit model comparison; EQCM-D immunosensor performance toward CD63-positive ESPs in HBS buffer (PDF)
