# Supplementary material for: Liquid Crystal-Templated Porous Microparticles via Photopolymerization of Temperature-Induced Droplets in a Binary Liquid Mixture
Source: ACS Omega. 2023 May 30;8(23):20404–11. doi: 10.1021/acsomega.3c00490 (PMC10268013; doi:10.1021/acsomega.3c00490)
Supplement: Supplementary file 1 — ao3c00490_si_001.pdf [file ao3c00490_si_001.pdf]

–SUPPLEMENTARY INFORMATION–

Liquid Crystal-Templated Porous Microparticles via Photopolymerisation of Temperature-Induced Droplets in a Binary Liquid Mixture

Mehzabin Patel,[†] Alberto Alvarez-Fernandez,[†] Maximiliano Jara Fornerod,[†] Anand N. P. Radhakrishnan,[†] Alaric Taylor,[†] Sing Teng Chua,[‡] Silvia Vignolini,[‡] Benjamin Schmidt-Hansberg,[¶] Alexander Iles,[§,‖] and Stefan Guldin[*,†]

†*Department of Chemical Engineering, University College London, London, WC1E 7JE, United Kingdom*

‡*Yusuf Hamied Department of Chemistry, University of Cambridge, Cambridge, CB2 1EW, United Kingdom*

¶*Chemical & Process Engineering, Coating & Film Processing, BASF SE, 67056 Ludwigshafen am Rhein, Germany*

§*Lab-on-a-Chip Research Group, Department of Chemistry and Biochemistry, University of Hull, Hull, HU6 7RX, United Kingdom*

‖*Now at: Department of Materials and Environmental Chemistry, Stockholm University, 106 91 Stockholm, Sweden*

E-mail: s.guldin@ucl.ac.uk

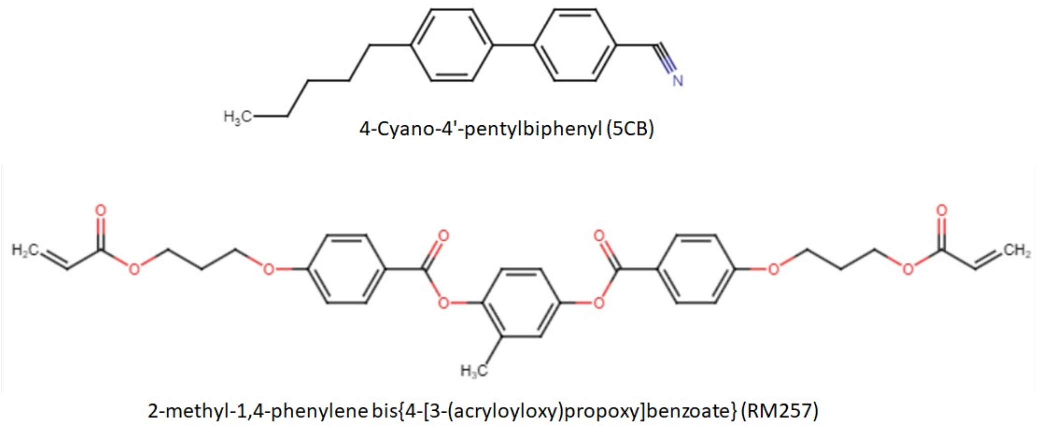

Supplementary Figure S1: Chemical structure of 4-cyano-4'-pentylbiphenyl (5CB) and 2-methyl-1,4-phenylene bis4-[3-(acryloyloxy)propoxy]benzoate (RM257)

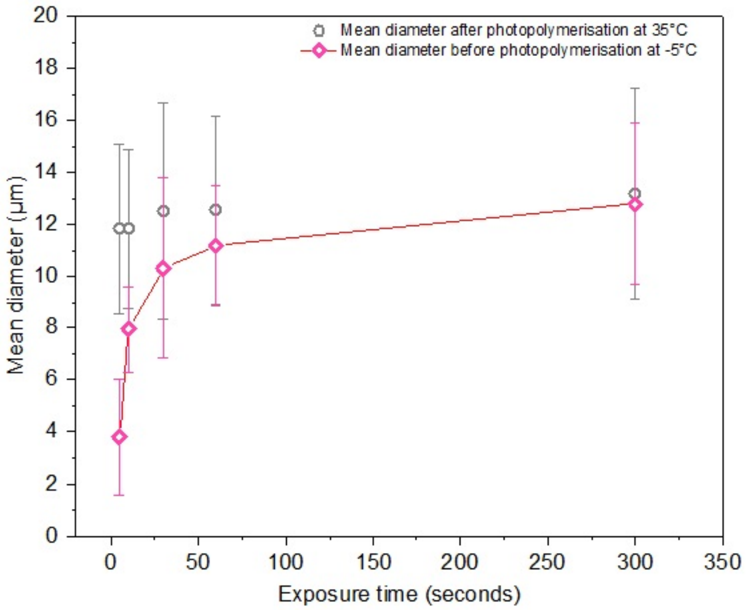

Supplementary Figure S2: Effect of UV light (365 nm) exposure time at 90mW cm^{-2} on shrinkage of droplets upon heating after photopolymerisation. The graph shows the mean diameter, with the error bars indicating the standard deviation for a sample size of 222 droplets

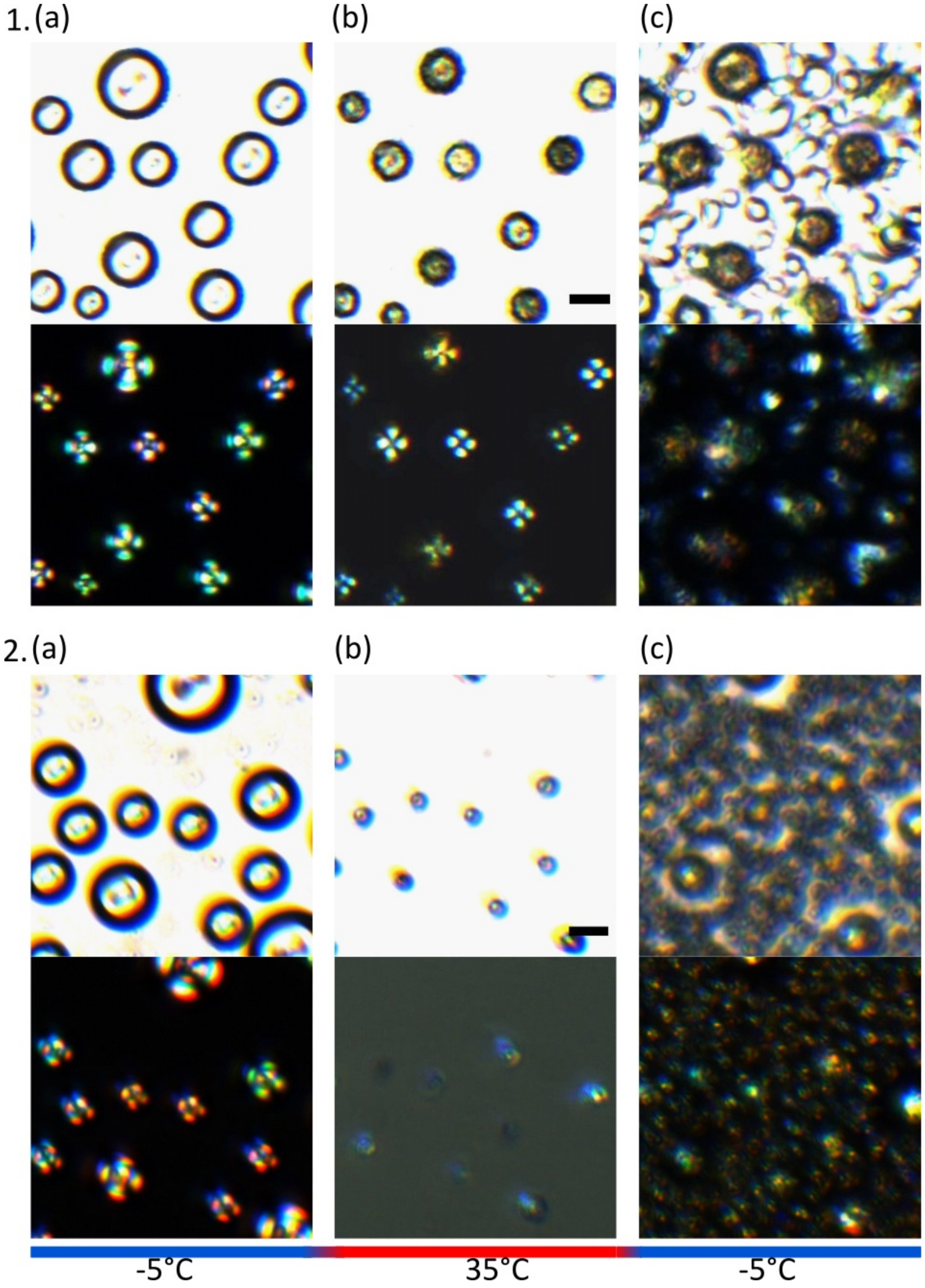

Supplementary Figure S 3: Microparticle shrinking and swelling after photopolymerisation. Particles exposed to UV light (3675nm) for (1) 1 minute and (2) 5 seconds. (1/2)a. 5CB/RM257 droplets at -5°C, immediately after UV exposure. (1/2)b. Liquid crystal templated microparticles after heating to 35°C. (1/2)c Swollen microparticles after cooling to -5°C, surrounded by newly nucleated droplets of 5CB. Images under Brightfield light (1 and 2. Top) and under crossed polarised light (1 and 2. Bottom). Scale:10 μm.

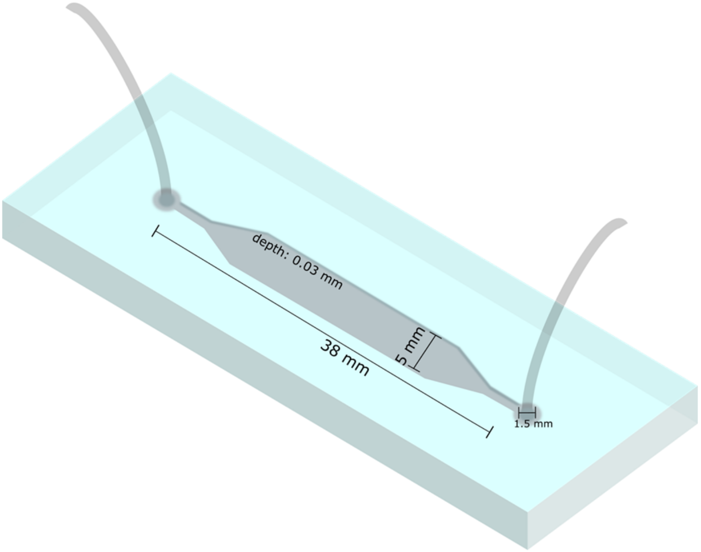

Supplementary Figure S4: Design and measurements of microfluidic chip chamber.

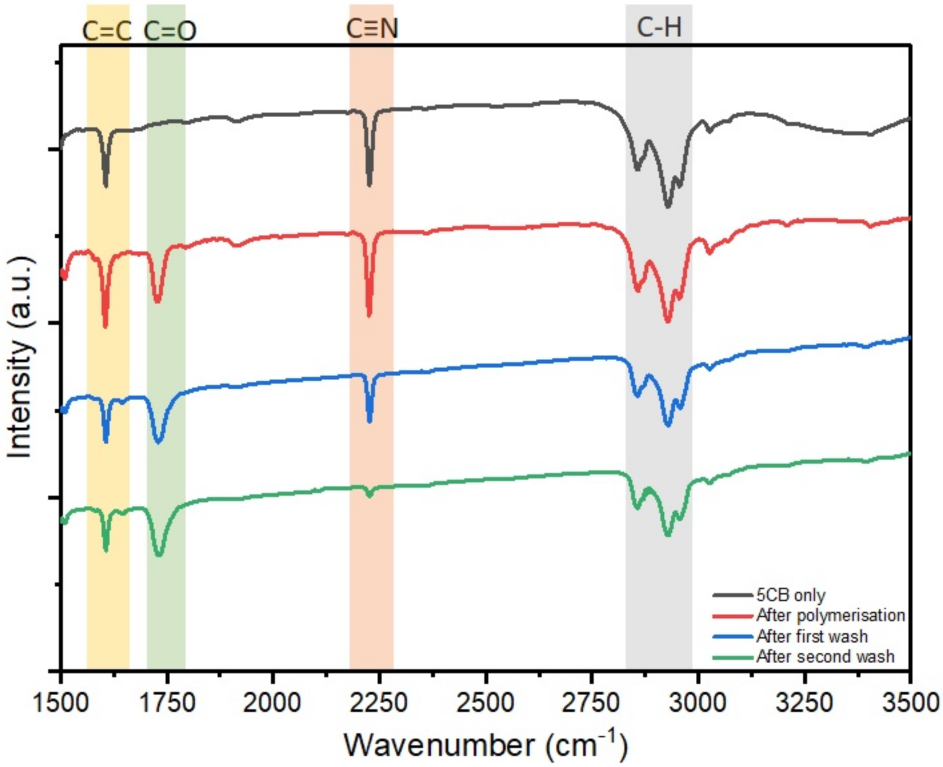

Supplementary Figure S 5: Infrared analysis of 5CB, 5CB/RM257 after photpolymerisation, and after washing by centrifugation and redispersion in acetone. The adsorption near 2250cm^{-1} is distinguised due to the C+N bond in 5CB. The adsorption at 1750cm^{-1} is characteristic of the C=O bond in RM257.

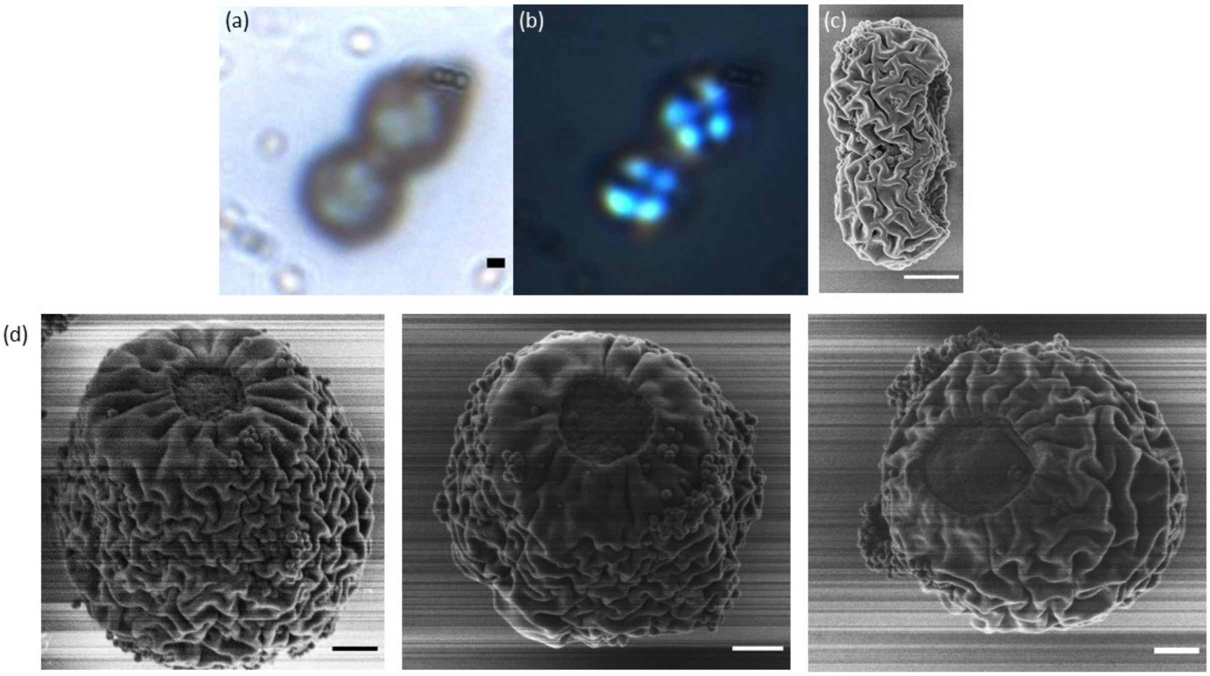

Supplementary Figure S6: Polymer microparticles. (a-c) Two particles fused together during photopolymerisation. The same particles shown after washing (a) under optical microscope, (b) under crossed polarisers, (c) with SHIM imaging. (d) More SHIM images polymer particles. Scale: 1 μm.
